# Differential, Phosphorylation Dependent Trafficking of AQP2 in LLC-PK1 Cells

**DOI:** 10.1371/journal.pone.0032843

**Published:** 2012-02-28

**Authors:** William L. Rice, Yan Zhang, Ying Chen, Toshiyuki Matsuzaki, Dennis Brown, Hua A. Jenny Lu

**Affiliations:** Program in Membrane Biology, Division of Nephrology, Center for Systems Biology, Department of Medicine, Massachusetts General Hospital and Harvard Medical School, Boston, Massachusetts, United States of America; University of Birmingham, United Kingdom

## Abstract

The kidney maintains water homeostasis by modulating aquaporin 2 (AQP2) on the plasma membrane of collecting duct principal cells in response to vasopressin (VP). VP mediated phosphorylation of AQP2 at serine 256 is critical for this effect. However, the role of phosphorylation of other serine residues in the AQP2 C-terminus is less well understood. Here, we examined the effect of phosphorylation of S256, S261 and S269 on AQP2 trafficking and association with recycling pathway markers. We used LLC-PK1 cells expressing AQP2(S-D) or (S-A) phospho mutants and a 20°C cold block, which allows endocytosis to continue, but prevents protein exit from the trans Golgi network (TGN), inducing formation of a perinuclear AQP2 patch. AQP2-S256D persists on the plasma membrane during cold block, while wild type AQP2, AQP2-S256A, S261A, S269A and S269D are internalized and accumulate in the patch. Development of this patch, a measure of AQP2 internalization, was most rapid with AQP2-S256A, and slowest with S261A and S269D. AQP2-S269D exhibited a biphasic internalization profile with a significant amount not internalized until 150 minutes of cold block. After rewarming to 37°C, wt AQP2, AQP2-S261A and AQP2-S269D rapidly redistributed throughout the cytoplasm within 20 minutes, whereas AQP2-S256A dissipated more slowly. Colocalization of AQP2 mutants with several key vesicular markers including clathrin, HSP70/HSC70, EEA, GM130 and Rab11 revealed no major differences. Overall, our data provide evidence supporting the role of S256 and S269 in the maintenance of AQP2 at the cell surface and reveal the dynamics of internalization and recycling of differentially phosphorylated AQP2 in cell culture.

## Introduction

Aquaporin-2 (AQP2) is critical for the maintenance of systemic water balance in mammals. Apical accumulation of AQP2 in renal collecting duct principal cells increases the permeability of the epithelium, allowing water to flow down its osmotic gradient into the interstitium and enter the circulation [1]–[4]. AQP2 is a constitutively recycled protein and is also subject to acute regulation. In the canonical pathway, this regulation is mediated via vasopressin (VP) signaling through its G coupled protein receptor (V2R) leading to increased intracellular cAMP, modulation of phosphorylation of AQP2 on the cytoplasmic COOH terminus (notably at serine 256 by protein kinase A (PKA)) accompanied by an increase in the rate of exocytosis [4]. As expected, compounds such as calcitonin [5] or prostaglandin E2 [6] that alter cAMP levels and/or the activity of PKA [7] are able to influence AQP2 phosphorylation and trafficking.

Recently, various non-canonical (i.e. non-VP mediated) pathways for AQP2 membrane accumulation have been demonstrated by our group and others. Phosphorylation of the AQP2 COOH terminus can be regulated by activation of protein kinase G (PKG) in response to elevated cGMP [8], [9]. Alteration of the polymerization of the actin cytoskeleton in the absence of VP stimulation can itself lead to the membrane translocation of AQP2 [10]–[12], and it is now well accepted that membrane accumulation of AQP2 can be achieved through the modulation of endocytosis and/or exocytosis [4], [13]–[19]. Inhibition of endocytosis by treatment with statins [17], [18], [20] or methyl-β-cyclodextrin [14], [16] results in the accumulation of AQP2 on the plasma membrane independent of phosphorylation. Furthermore, we have observed that VP stimulation increased rates of exocytosis even in cells expressing an AQP2 mutant (AQP2-S256A) that cannot be phosphorylated at serine 256 [15]. Therefore, although a role for PKA/PKG mediated AQP2-S256 phosphorylation in inhibiting AQP2 endocytosis has been clearly demonstrated [21], [22], its influence on AQP2 exocytosis is less certain, partially due to the difficulty of separating the endocytosis and exocytosis pathways and the constant, rapid recycling of AQP2 [14].

Phospho-proteomic studies [23], [24] have identified S261, S264 and S269 as additional residues with phosphorylation states that are modulated by VP. While the role of phosphorylation at these residues is not fully understood, emerging data suggest that differential phosphorylation at these sites can also regulate the trafficking of AQP2. For example, S261 is de-phosphorylated in response to VP treatment [25], and pS261 is found mostly in intracellular vesicles after ubiquitination and endocytosis indicating a possible role stabilizing intracellular AQP2 localization [23], [25], [26]. On the other hand, phosphorylation at S269 has been detected only on the plasma membrane [24], and recent data from polarized MDCK cells expressing AQP2-S269D indicates that pS269 conveys a resistance of AQP2 to endocytosis [22]. However, it has also been shown that the S256 residue seems to be the “master switch” whose phosphorylation is necessary for downstream phosphorylation of other C-terminal serines [21], [24]. In addition, we have shown previously that phosphorylation at S256 is not necessary for AQP2 recycling, because AQP2-S256A recycles rapidly and constitutively [14]. The role of phosphorylation at the other C-terminus serine residues in AQP2 recycling remains to be fully resolved.

Therefore, in this study we set out to investigate the role of AQP2 phosphorylation sites on non-stimulated (constitutive recycling) endocytosis and exocytosis using AQP2 with point mutations that mimic either the phosphorylated or un-phosphorylated state of serine 256, 261 and 269. We are able to isolate the endocytotic and exocytotic pathways by employing the “cold block” and “cold block release” methods to follow AQP2 trafficking. As observed nearly twenty years ago, the transport of membrane proteins from the Golgi to the plasma membrane can be blocked by incubating cells at 20°C, inhibiting the exit of protein from the Golgi [27]. Since then, membrane protein recycling has been studied by applying the cold blocking method to interrupt the recycling of internalized membrane proteins and cause their accumulation in the trans Golgi network (TGN). When followed with a cold block release by rewarming to 37°C, the rapid re-initiation of protein trafficking from TGN to plasma membrane can be followed. We have successfully applied this cold block approach previously in our work on both WT-AQP2 and V2R trafficking [28], [29]. In addition to assessing the internalization of AQP2, we examined the colocalization of the water channel with markers of the recycling pathway to determine if changes in AQP2 phosphorylation affected the association with these subcellular compartment markers.

## Methods

### Antibodies and chemicals

All chemicals unless otherwise noted were purchased from Sigma-Aldrich (St. Louis MO), and cell culture reagents were obtained from Invitrogen (Carlsbad CA). Monoclonal antibodies against c-myc were generated from the 9E10 hybridoma cell line, purchased from American Type Culture Collection (ATCC, Manassas VA). Secondary antibodies tagged with either Cy3, CY5.5 or FITC were obtained from Jackson Immunoresearch Laboratories (West Grove PA). The following commercial primary antibodies were purchased from their respective vendors: EEA1 (Cell Signaling 2411s Danvers MA), Rab5 (Cell Signaling 2143s), Rab7 (Cell Signaling 2094s), Rab10 (Sigma-Aldrich R8906), HSP70/HSC70 (Abcam ab53496, Cambridge MA), Lysotracker Red (Invitrogen), Clathrin (BD Transduction Labs 610499, Franklin Lakes NJ). LLC-PK1 cells were purchased from ATCC (CL-101).

### AQP2 point mutations and generation of stable cell lines

Point mutations substituting either an aspartic acid (D) or alanine (A) for serine (S) on the C-terminus of AQP2 at positions 256, 261 and 269 were generated using site directed mutagenesis (Invitrogen) as previously reported [21]. The following primers were used: S256A: (F) 5′ GTGCGGCGGCAG**GCA**GTGGAG 3′, S256A: (R) 5′ GCCGTC**CGT**CACCTCGAGGTGAGA 3′. S256D: (F) 5′ GTGCGGCGGCAG**GAC**GTGGAG 3′ S256D: (R) 5′ GCCGTC**CTG**CACCTCGAGGTGAGA 3,′ S261A: (F) 5′ GGTGGAGCTCCAC**GCT**CCTCAGAGCC 3′, S261A: (R) 5′ GGCTCTGAGG**AGC**GTGGAGCTCCACC 3′. S269A: (F) 5′ GAGCCTGCCTGCCGGC**GCC**AAGGCCGAACAAAAGC 3′, S269A: (R) 5′ GCTTTTGTTCGGCCTT**GGC**GCCGCGAGGCAGGCTC 3′. S269D: (F) 5′ GAGCCTGCCTGCCGGC**GAC**AAGGCCGAACAAAAGC 3′, S269D: (R) 5′ GCTTTTGTTCGGCCTT**GTC**GCCGCGAGGCAGGCTC 3′. Stable cell lines expressing these phospho-mimic AQP2 mutants were obtained by transfection of the porcine kidney cell line, LLC-PK1, using Lipofectamine (Invitrogen) and selection under 500 µg/mL G418. All cell lines were maintained in DMEM supplemented with 10% bovine serum and 1% penicillin/streptomycin at 37°C in a humidified 5% CO_2_ atmosphere.

### Cold block

LLC-PK1 - AQP2 cells were plated on 15×15 mm glass cover slips (Electron Microscopy Sciences) at least 24 hours before experimentation. To inhibit newly synthesized protein production, cycloheximide was added to the culture medium (10 µg/mL) for 60 minutes prior to, and maintained in the medium during cold block. Cold block was performed by placing the culture plates at 20°C in a water bath for various time points up to 150 minutes. Cells were then fixed with 4% paraformaldehyde/PBS (PH 7.4) and subjected to immunofluorescence staining. For the cold block and release experiment, after cold block for 2 hours, which gives the maximal perinuclear patch structure, cells were brought to 37°C to allow protein/vesicle recycling to occur. Cells were harvested at various time points after cold block release, fixed and processed for immunofluorescence staining.

### Immunofluorescence staining

AQP2 and subcellular markers were localized by immunofluorescence staining via a standard protocol. Cells were permeabilized in 0.01% Triton X-100 in PBS for 4 minutes, blocked with a 1% BSA/PBS for 20 minutes and then incubated with primary antibody overnight at 4°C. After washing with PBS, cells were incubated with the secondary antibodies at room temperature for one hour. Subsequently, the cover slips were washed and mounted in Vectashield containing DAPI (Vector Labs., Burlingame, CA) and visualized on a Nikon 80i microscope with a ORCA95 camera (Hamamatsu, Tokyo Japan). For quantitative analysis of AQP2 trafficking, all cover slips were stained under the same conditions and imaged with the same microscope parameters. Analysis of the AQP2 patch fluorescence was performed using IPLAB software (Biovision, Exton PA). The region of interest used to measure AQP2 patch fluorescence was determined by applying an intensity threshold to the visible perinuclear accumulation of AQP2 (Figure S1). Colocalization of AQP2 with markers of the recycling pathway was calculated from a series of confocal sections (1 µm step size) acquired on a Nikon A1 confocal microscope (Nikon, Tokyo Japan) using Volocity software (Perkin Elmer, Waltham MA) (Figure S2). For publication, image brightness and contrast were linearly adjusted, and a high pass filter was applied to remove noise in Adobe Photoshop (Adobe, San Jose CA).

### Statistics

Statistics were performed with the Prism software (GraphPad, La Jolla CA). Data for each treatment (cold block or cold block release) were first compared for significance with a one-way ANOVA. Differences in means were then compared between AQP2 mutants at each time point using the student's T-test (two tailed). Each experiment was repeated at least three times. Statistical significance was determined at a p value<0.05.

## Results and Discussion

### Stable cell lines expressing various AQP2 phosphorylation mutants

**Figure 1:**
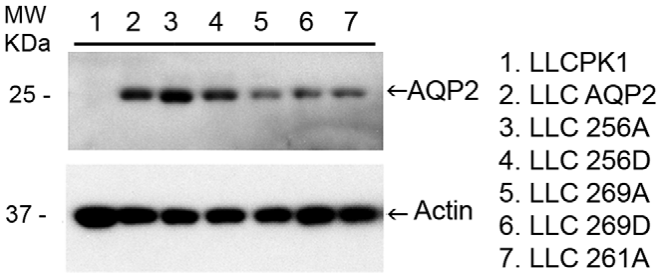
Expression of AQP2 and various phosphorylation mutants in LLCPK1 cells. Cell lysates were prepared from stable cells expressing WT AQP2 or AQP2 phosphorylation mutants, including S256A, S256D, S261A, S269A and S269D. Using an antibody recognizing the myc tag that is attached to the C-terminus of AQP2, AQP2 appears as an approximately 25 kDa band on western blot.

Stable expression of AQP2 phosphorylation mutants containing a c-myc tag fused to the COOH terminus was achieved in LLC-PK1 cells. Based on the rat AQP2 sequence, point mutations at serine residues 256, 261, or 269 were created substituting either aspartic acid (D), mimicking the charged state of phosphorylation, or alanine (A), which mimics the de-phosphorylated state (Identified as: S256D, S256A, S261A, S269D, S269A). AQP2 expression in these cell lines was confirmed by western blot using an antibody against the c-myc tag and a commercially available polyclonal goat anti-AQP2 antibody recognizing AQP2 C-terminus (Santa Cruz, CA). Both antibodies showed similar results and the immunoblot using anti-c-myc antibody is presented here (Fig. 1).

### Effect of phosphorylation on AQP2 endocytosis

**Figure 2:**
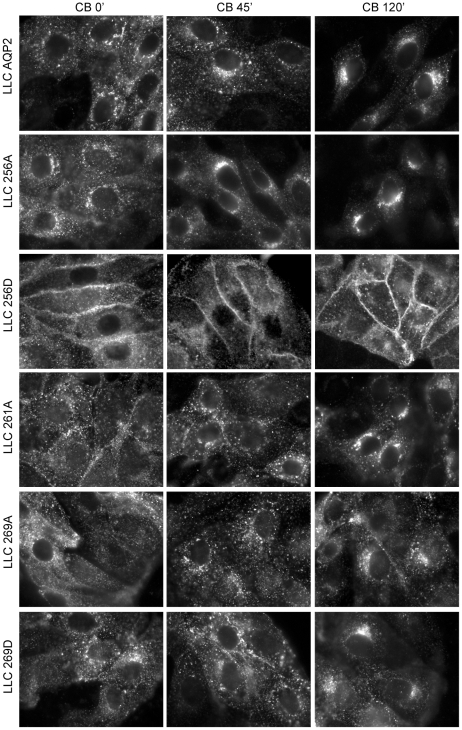
Formation of AQP2 perinuclear patch after cold block at 20°C in cells expressing various AQP2 phosphorylation mutants. After pretreatment with cycloheximide for one hour, cells underwent cold block at 20°C for up to 150 minutes. Cells were fixed and subjected to staining with anti-c-myc antibody to detect AQP2. During 120 minutes of cold block the development of the perinuclear AQP2 patch is evident in all cells expressing AQP2 variants except for AQP2-S256D, which remains mainly on the cell membrane (LLC 256D). Scale bar = 20 µm.

To study the role of phosphorylation of AQP2 on its endocytosis, LLC-PK1 cells expressing the AQP2 constructs were incubated at 20°C (cold block) for up to 150 minutes and evaluated by immunofluorescence staining with anti-c-myc antibodies as previously described [21]. During the cold block, the gradual formation of a perinuclear patch was evident for all of the AQP2 phospho-mutants except for AQP2-S256D, the majority of which remained on the cell membrane (Fig. 2). The perinuclear patch consists of internalized AQP2 that is sequestered at the level of the trans Golgi network by incubation of the cells at 20°C, which has been shown to prevent protein from exiting the TGN [29] and which inhibits the constitutive recycling of transferrin receptor [30], [31]. The contribution of newly synthesized AQP2 to the perinuclear patch was minimized by the inhibition of *de novo* protein synthesis with cycloheximide in the culture medium. Cycloheximide at this concentration has been previously shown by our group to significantly inhibit AQP2 and V2R synthesis [28], [32]. Our initial experiment of cold block treatment of mutant cells for 2 hours in the presence or absence of cycloheximide revealed a similar AQP2 signal intensity with or without cycloheximide suggesting that a significant contribution of the perinuclear signal from newly synthesized AQP2 is unlikely (data not shown). In addition, the half life of AQP2 at 37°C has been reported to be between 6 and 12 hours in mpkCCD (c14) [33] and MDCK cells [34]. Therefore, the majority of the AQP2 accumulating in the patch during the 2 hours of cold block is due to recycled protein, and the contribution of protein degradation is likely to be minimal.

**Figure 3:**
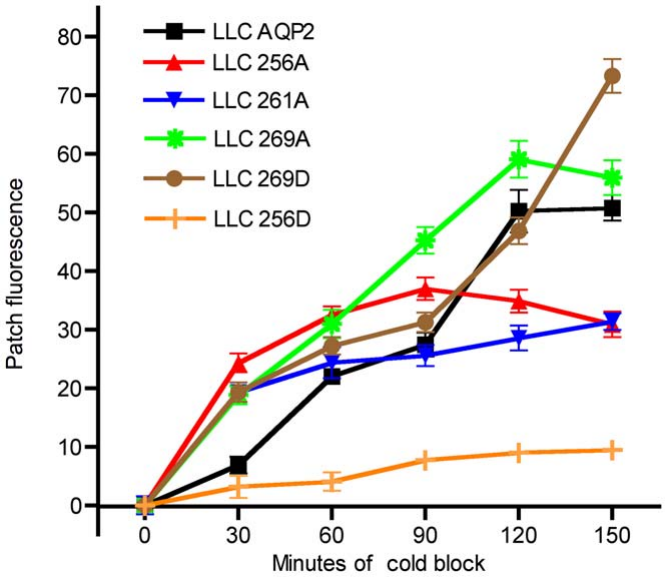
Quantified formation of AQP2 perinuclear patch after cold block at 20°C in cells expressing various AQP2 phosphorylation mutants. The time course of the development of the AQP2 perinuclear patch was quantified by measuring mean pixel intensity of the patch using IPlab software. The results are presented as the increase in mean patch fluorescence value starting from 0 minutes of cold block. The overall accumulation of the perinuclear patch reached a maximal density after cold block for 150 minutes with all mutants. The experiment was repeated in triplicate, N for each mean is ≥24. Bars represent standard error.

To quantify the rate of accumulation of endocytosed AQP2, the formation of the perinuclear patch was evaluated by immunofluorescence staining every 30 minutes over 150 minutes of cold block at 20°C. Representative images for each AQP2 mutant at 0, 45 and 120 minutes of cold block are displayed in Figure 2. The quantification of fluorescence images representing the time dependent development of the perinuclear AQP2 patch is shown in Figure 3. Consistent with previous reports [21], [22], [35], [36], we found a persistent membrane presence of AQP2-S256D with dramatically reduced endocytosis (patch formation) during cold block compared to the other AQP2 mutants, suggesting that phosphorylation at S256 leads to a resistance to endocytosis. Conversely, the S256A mutant was not retained on the plasma membrane and internalized rapidly after cold block with no further significant increase in perinuclear patch fluorescence after it reached a maximum intensity at 60 minutes (P<0.5). In contrast, maximum perinuclear fluorescence in LLC-AQP2 and LLC-S269A cells was achieved at either 120 minutes or 150 minutes of cold block for the S261A and S269D AQP2 mutants. Interestingly, the S269D mutant displayed a biphasic internalization, initially reaching a patch fluorescence intensity of 37% of maximum by 60 minutes which plateaus until 120 minutes. Compared to the wild type AQP2, both S261A and 269A displayed a rapid initial internalization at 30 minutes followed by a steady subsequent growth of the patch.

### Exocytosis and recycling after cold block release

**Figure 4:**
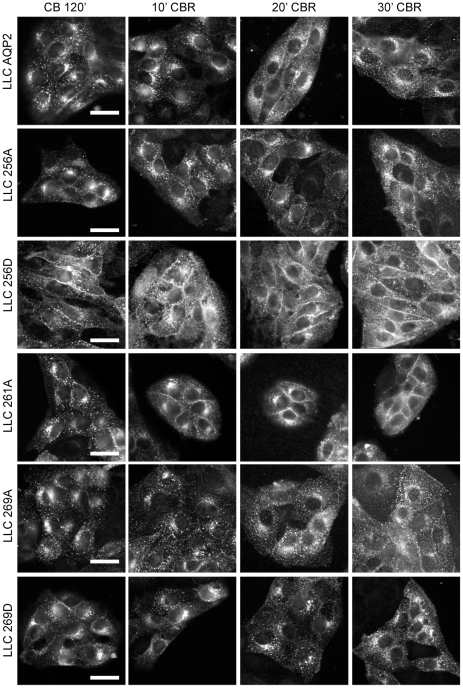
Dissolution of the AQP2 perinuclear patch after cold block release. Cells were cold blocked for 120 minutes to form the perinuclear patch after which the cold block was released by returning the cells to 37°C. The perinuclear patch rapidly disintegrated over 30 minutes of cold block release as the AQP2 mutants (except for AQP2-S256D which remained on the membrane) redistributed throughout the cytosol and membrane. Scale bar = 20 µm.

**Figure 5:**
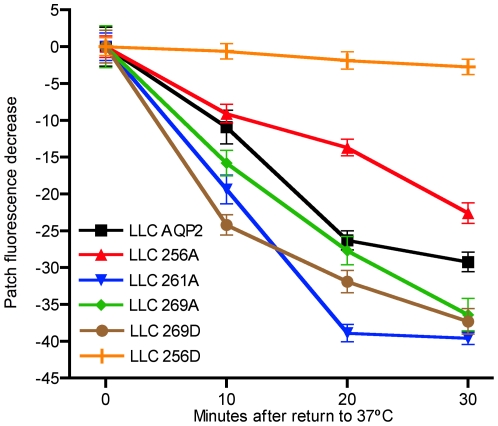
Quantified dissolution of the AQP2 perinuclear patch after cold block release. To quantify the rate of AQP2 redistribution a time course analysis of the dissolution of the patch was assayed by immunofluorescence staining and images were quantified using the IPlab software. The results are presented as the decrease from the maximum mean patch fluorescence value after release of cold block. The experiment was repeated in triplicate N for each mean is > = 24. Bars represent standard error.

To investigate the impact of the phosphorylation mutations on the exocytosis of AQP2, cells were returned to 37°C after 120 minutes of cold block at 20°C. In Figure 4 the dissolution of the perinuclear AQP2 patch is evident by 30 minutes of cold block release. As observed during the cold block, AQP2-S256D is primarily membrane bound, while the other AQP2 phosphorylation mutants redistributed throughout the cytoplasm. To compare the relative rates of exocytosis, the dissolution of the perinuclear patch was assessed by immunofluorescence staining every 10 minutes over 30 minutes of cold block release. In Figure 5, the decrease in mean AQP2 perinuclear patch fluorescence is compared. As was seen during the cold block, little change in the already low intensity of AQP2-S256D perinuclear fluorescence was observed. The perinuclear patch in LLC S256A, S269A and S269D cells reached minimum fluorescence intensities after 30 minutes. On the other hand, wt AQP2 and S261A patches resolved by 20 minutes of cold block release. In contrast to its relatively rapid internalization during cold block, the AQP2-S256A perinuclear patch was the slowest to dissolve upon returning the cells to 37°C.

### AQP2 colocalization with markers of vesicular compartment

AQP2 is a constitutively trafficked protein that is recycled from the plasma membrane beginning in clathrin coated pits [37], into EEA1 positive vesicles and sorted to either lysosomes for degradation or through the TGN and back to the plasma membrane. Therefore, in addition to investigating the rate of AQP2 internalization during cold block and subsequent exocytosis following cold block release, we examined whether phosphorylation of AQP2 impacted its association with markers of key subcellular compartments including clathrin, HSP70/HSC70, EEA1, Rab7, Rab10, Lysotracker and TGN proteins.

**Figure 6:**
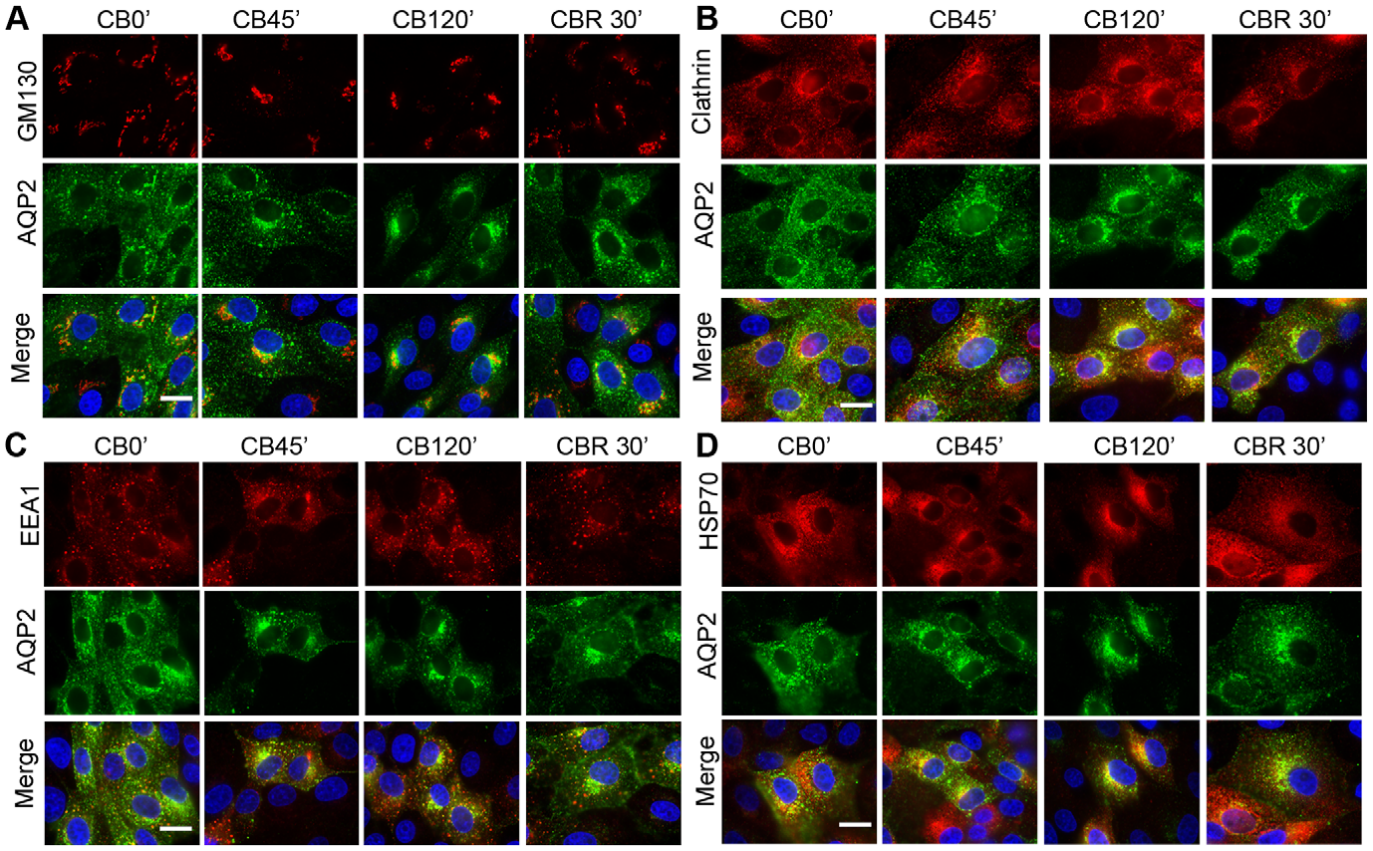
Association of AQP2 and cellular compartment markers during the dynamic process of cold block and cold block release. The association of subcellular compartment markers with AQP2 during the cold block and subsequent release was investigated. GM130, clathrin, EEA1, and HSP/HSC70, were co-stained with AQP2 (A, B, C, D). Panel A, GM130 staining also overlaps with AQP2 in the perinuclear patch but not after release of the cold block. In panel B, clathrin appeared to colocalize mostly with AQP2 during the development of the perinuclear patch and redistribution after cold block release. Panel C, EEA staining was colocalized with AQP2 at the early stage of endocytosis, and was partially associated with the perinuclear patch. After CBR at 37°C, a few large EEA/AQP2 positive structures appeared, but most AQP2 was not associated with EEA. Panel D, HSP/HSC70 partially colocalized with AQP2 during the formation of the perinuclear patch, but not during AQP2 redistribution after cold block release. Scale bar = 20 µm.

In Figure 6 we show double immunofluorescence labeling of LLC-PK1 cells expressing wild type AQP2 with TGN, HSP70/HSC70, clathrin, or EEA1 during cold block and cold block release. For each of these markers, visual inspection of the samples revealed no obvious differences in immunofluorescence colocalization among the AQP2 mutants, except for the internalization resistant AQP2-S256D. Quantitative analysis of colocalization of each of these markers and the AQP2 mutants did not provide any additional significant insights into the role of phosphorylation on AQP2 compartmentalization during constitutive trafficking (Figure S2). However some interesting patterns of interaction were observed in the data among the mutants during cold block and release. For example an increase in colocalization of AQP2 S256A and GM130 was noted during the cold block, and persisted during the cold block release suggesting a delayed transit from the TGN to recycling vesicles, which is consistent with reports in the literature [38].

Cold block of LLC-PK1 cells inhibits protein exit from the TGN and results in a condensation of AQP2 staining in a perinuclear region that is also labeled by the TGN protein GM130 (Figure 6 A). In agreement with previous studies, the AQP2 variants were found to colocalize with clathrin and HSP70/HSC70 within the perinuclear patch [29]. While the majority of AQP2-S256D remained on the plasma membrane, which may account for our previous observation of reduced interaction of HSP70 and this mutant [22], [39], some colocalization of HSP70/HSC70 and the small fraction of internalized AQP2-S256D was observed (Figure S2).

Association with EEA1 decreased during the cold block, with a subsequent increase following cold block release (Figure 6 C and Figure S2). Rab7- AQP2 colocalization was observed mainly within the already formed patch. Interestingly, little to no colocalization of AQP2 and Rab10 or Lysotracker was observed at any stage (not shown) despite reports in the literature that AQP2 is found in Rab10 positive vesicles by LM-MS [40] and that AQP2-S256A is prominent in lysosomes [38]. These data indicate that, aside from pS256, phosphorylation at these residues does not seem to significantly alter the internalization path or major subcellular compartments in which AQP2 resides during endocytosis and recycling. Further examination with subcellular fractionation in combination with biochemical characterization may be needed to dissect the vesicular pathway in association with the differential phosphorylation of AQP2 along its trafficking pathways.

The current model of regulated AQP2 trafficking links VP stimulated phosphorylation of serine 256 with membrane accumulation of the water channel [41]. *In vivo*, VP stimulation leads to dramatic apical membrane staining of AQP2 with antibodies specific for phosphorylation at serine 256 (pS256), and semi-quantitative analysis by western blot reveals an increase in the percentage AQP2 with S256 phosphorylation. *In vitro*, the dominant role of S256 in membrane accumulation is supported by the constitutive membrane presence of AQP2 in cells expressing the phospho-mimic mutation AQP2-S256D, an effect that is not altered by S-D or S-A substitutions at S261, or S269 [21]. Endocytosis of AQP2-S256D may however be modulated *in vitro* by ubiquitination at K270 following the addition of 12-tetradecanoylphorbol-13-acetate (TPA) [26], [35].

In contrast, AQP2-S256A remains mostly cytosolic even during stimulation of cells by VP and/or forskolin. The S256A mutation, however, does not prevent the constitutive recycling of the water channel, and membrane accumulation of AQP2 S256A can be readily observed upon acute inhibition of endocytosis [14]. Surprisingly, expression of the S256A mutation does not result in an inhibition of stimulated exocytosis of AQP2 after treatment of cells with VP [15]. Even though the functional significance and regulation, if any, of the constitutive recycling of AQP2 is not yet understood, current data suggest that in contrast to VP mediated AQP2 trafficking, constitutive recycling of AQP2 does not depend on the phosphorylation of S256. A recent report has shown that AQP2-S256D has a reduced association with endocytotic proteins, such as clathrin, dynamin and HSC70 in MDCK cells [22]. Therefore, it is likely that phosphorylation at S256 plays a more dominant role in the maintenance of AQP2 membrane presence via resistance to endocytosis rather than being directly responsible for stimulated trafficking and/or exocytosis. However, one study has suggested that S256D can be internalized after treatment of cells with dopamine, but only after cells were first pre-exposed to forskolin [35]. This was interpreted as reflecting the need for FK-induced phosphorylation of as yet unknown components of the endocytotic machinery to facilitate AQP2 endocytosis.

We quantified some significant differences among the AQP2 mutants in the rate at which the perinuclear patch accumulated. The most rapid accumulations were seen with the S256A and S261A mutants. This is consistent with the fact that the S256A mutant cannot be phosphorylated at the critical S256 site, and its transit through the plasma membrane is not retarded, as could be the case for at least some of the wild type protein, which may have some level of constitutive S256 phosphorylation. The greater speed of S261A accumulation compared to the wild type protein is more difficult to rationalize, since it too could have some baseline level of S256 phosphorylation. Our data indicate that, at the very least, VP-induced dephosphorylation of S261 is not by itself a signal for cell surface retention of AQP2.

The role of the S269 site is slowly emerging. pS269 is observed only on the apical membrane *in vivo*, and a role in retarding endocytosis has been suggested [22], [24]. Our present data support this hypothesis, since the S269D mutation was internalized much more slowly than S269A. In our artificial AQP2-S269D expression system, intracellular vesicles containing the mutant are clearly detectable, whereas *in vivo*, antibodies against pS269 label only the plasma membrane [24], [42]. We found a biphasic pattern of growth of the perinuclear patch in our cell system. One intriguing possibility is that the initial growth, which plateaus between 50–100 minutes of cold treatment, may reflect the recycling of these intracellular vesicles back to the TGN. The later accumulation, beginning about 100 minutes after cold exposure, may reflect the retarded internalization of an “endocytosis resistant” pool of membrane bound S269D that was either in the plasma membrane initially, or in the final stages of the constitutive insertion pathway prior to cold exposure. We have shown previously that endocytosis from the cell surface contributes to patch formation, but we cannot rule out the possibility that – as we suggest for the S269D mutation – there is at least some contribution from retrograde transport of vesicles that are already present inside the cell at the time of cold exposure.

Upon re-warming of cells to 37°C, the tight perinuclear patch containing AQP2 begins to disperse, as material is released from the cold-induced TGN export block. It has been shown for AQP2, as well as in other systems, that this released material can enter the secretory pathway, and be inserted into the plasma membrane through constitutive vesicle exocytosis [29]. In addition, we have shown previously that cAMP is not elevated following the release of cold block and, thus, it is unlikely that the rate of patch dissolution is dependent on stimulated exocytosis [29]. In addition, the endogenous level of cAMP was not altered in these stable cell lines expressing these AQP2 phosphorylation mutants (data not shown). We found that while three of the AQP2 constructs behaved similarly after re-warming (WT, S216A and S269A), dissipation of the S256A containing patch was significantly slower than for the other AQP2 constructs and that of the S269D mutation was more rapid. It has previously been suggested that phosphorylation of S256 is somehow involved in AQP2 transport and processing in the recycling pathway, and that release into the secretory pathway is inhibited by dephosphorylation [38]. Our past and present data show that while S256A seems to follow a similar trafficking itinerary to the wild type protein, its transit through and/or release from the TGN may indeed be slower compared to wild type AQP2 and the other phosphorylation mutants. It appears that phosphorylation of the S269 residue may, in contrast, increase the release of AQP2 from the TGN, although whether regulation occurs *in vivo* remains to be determined.

While the differential role of various AQP2 phosphorylation sites on AQP2 regulated trafficking as well as constitutive recycling will continue to be the subject of future studies, our data demonstrate that differential phosphorylation of AQP2 affects the rate and pattern of endocytosis and recycling of AQP2 in cells.

## Supporting Information

Figure S1 **Region of Interest around perinuclear patch.** The region of interest (yellow line) used to measure AQP2 patch fluorescence was determined by applying an intensity threshold to the visible perinuclear accumulation of AQP2.
(TIF)

Figure S2 **Quantification of colocalization between AQP2 and markers of the subcellular compartment.** The fraction of AQP2 signal colocalized with each subcellular compartment marker is presented for 0, 45, and 120 minutes of cold block as well as for 30 minutes following cold block release. For each data set, the colocalizations were calculated from multiple individual cells in confocal microscope stacks taken at a step size of 1 µm. Means are presented with standard error bars. Despite the formation of a tight perinuclear patch in the same vicinity as the Golgi apparatus, as can be observed in Figure 6, only a small fraction (generally sub 15%) of AQP2 is colocalized on a vesicular level with the TGN marker GM130. Specifically, for GM130 staining, a significant colocalization of AQP2 and GM130 is seen in the LLC 256A mutant before the cold block and is increased after cold block. Cold block release does not change the fraction of association suggesting a delayed dissociation or a stable association of AQP2 with GM130 even after cold block release. Minimal association of AQP2 and GM130 is seen in LLC 269A and LLC 269D. Of the very small subpopulation of AQP2-S256D that gets internalized, AQP2 staining colocalizes with GM130 after cold block and cold block release. For clathrin staining, an increasing association with clathrin signal during cold block was observed followed by a decreased association during cold block release in the mutants except for LLC 269A which experienced an increased AQP2:clathrin colocalization only after cold block release. For EEA staining, AQP2 has a generally low degree of association with EEA and no particular pattern is suggested by the quantification. For HSP/HSC70 staining, initial high level of colocalization of AQP2 and HSP/HSC70 was seen in the early endocytosis phase in wild type, LLC 256A and LLC 256D, and is reduced after cold block 45 minutes, while it is increased for LLC 261A, LLC 269A and LLC 269D. A biphasic pattern of AQP2/HSP/HSC70 colocalization during the cold block is observed for LLC 261A, LLC 269A, and LLC 269D. Interestingly, AQP2-S261A, -S269A and -S269D show increased colocalization of HSP/HSC70 and AQP2 during “cold block release”, while wild type, -S256A, and -S256D do not.
(PDF)
