# Supplementary figures and images for: Differential, Phosphorylation Dependent Trafficking of AQP2 in LLC-PK1 Cells
Source: PLoS One. 2012 Feb 28;7(2):e32843. doi: 10.1371/journal.pone.0032843 (PMC3293519; doi:10.1371/journal.pone.0032843)

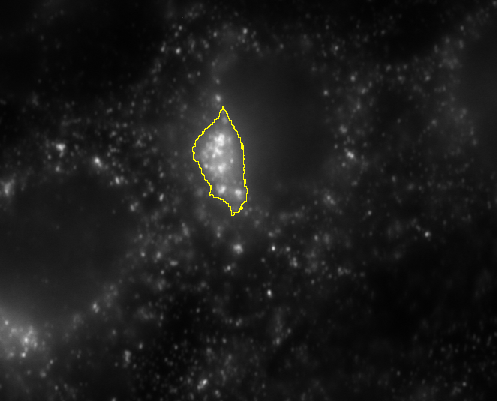

Supplement: Figure S1 — Region of Interest around perinuclear patch. The region of interest (yellow line) used to measure AQP2 patch fluorescence was determined by applying an intensity threshold to the visible perinuclear accumulation of AQP2. (TIF) [file pone.0032843.s001.tif]

**AQP2:GM130**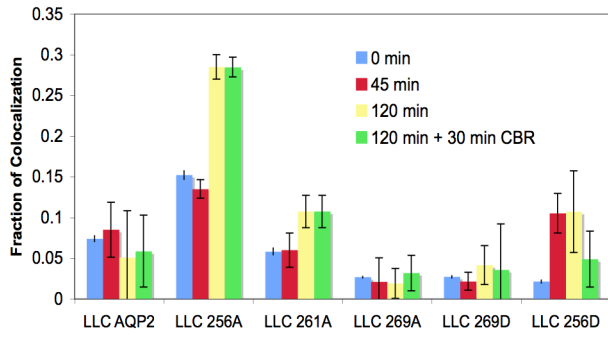**AQP2:Clathrin**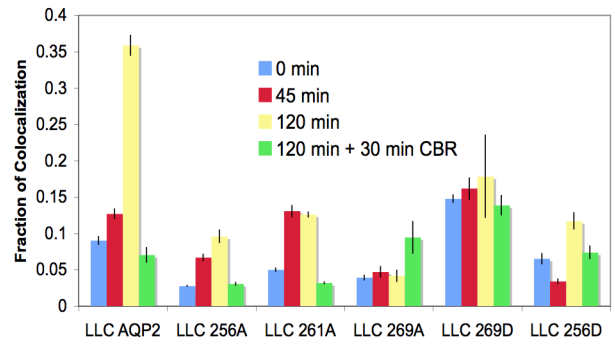**AQP2:EEA1**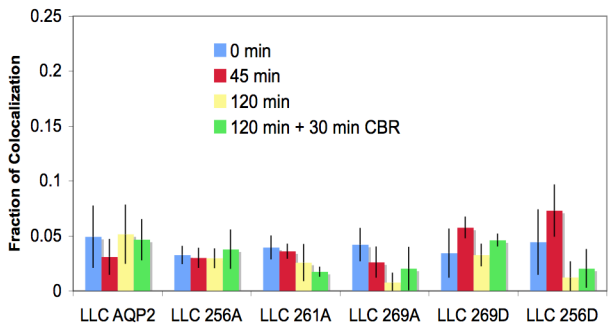**AQP2:HSP/HSC70**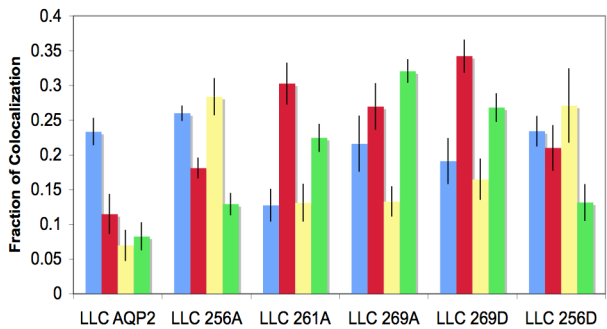

Supplement: Figure S2 — Quantification of colocalization between AQP2 and markers of the subcellular compartment. The fraction of AQP2 signal colocalized with each subcellular compartment marker is presented for 0, 45, and 120 minutes of cold block as well as for 30 minutes following cold block release. For each data set, the colocalizations were calculated from multiple individual cells in confocal microscope stacks taken at a step size of 1 µm. Means are presented with standard error bars. Despite the formation of a tight perinuclear patch in the same vicinity as the Golgi apparatus, as can be observed in Figure 6, only a small fraction (generally sub 15%) of AQP2 is colocalized on a vesicular level with the TGN marker GM130. Specifically, for GM130 staining, a significant colocalization of AQP2 and GM130 is seen in the LLC 256A mutant before the cold block and is increased after cold block. Cold block release does not change the fraction of association suggesting a delayed dissociation or a stable association of AQP2 with GM130 even after cold block release. Minimal association of AQP2 and GM130 is seen in LLC 269A and LLC 269D. Of the very small subpopulation of AQP2-S256D that gets internalized, AQP2 staining colocalizes with GM130 after cold block and cold block release. For clathrin staining, an increasing association with clathrin signal during cold block was observed followed by a decreased association during cold block release in the mutants except for LLC 269A which experienced an increased AQP2:clathrin colocalization only after cold block release. For EEA staining, AQP2 has a generally low degree of association with EEA and no particular pattern is suggested by the quantification. For HSP/HSC70 staining, initial high level of colocalization of AQP2 and HSP/HSC70 was seen in the early endocytosis phase in wild type, LLC 256A and LLC 256D, and is reduced after cold block 45 minutes, while it is increased for LLC 261A, LLC 269A and LLC 269D. A biphasic pattern of AQP2/HSP/HSC70 c [file pone.0032843.s002.pdf]
